# The α-tubulin acetyltransferase ATAT1: structure, cellular functions, and its emerging role in human diseases

**DOI:** 10.1007/s00018-024-05227-x

**Published:** 2024-04-23

**Authors:** Angela Iuzzolino, Francesca Romana Pellegrini, Dante Rotili, Francesca Degrassi, Daniela Trisciuoglio

**Affiliations:** 1 IBPM Institute of Molecular Biology and Pathology, CNR National Research Council of Italy, Via degli Apuli 4, Rome, 00185 Italy; 2 Department of Drug Chemistry & Technologies, Sapienza University of Rome, Piazzale Aldo Moro 5, Rome, 00185 Italy

**Keywords:** Cytoskeleton, Microtubules, Tubulin acetylation, Acetyltransferase, Neurological diseases, Cancer

## Abstract

The acetylation of α-tubulin on lysine 40 is a well-studied post-translational modification which has been associated with the presence of long-lived stable microtubules that are more resistant to mechanical breakdown. The discovery of α-tubulin acetyltransferase 1 (ATAT1), the enzyme responsible for lysine 40 acetylation on α-tubulin in a wide range of species, including protists, nematodes, and mammals, dates to about a decade ago. However, the role of ATAT1 in different cellular activities and molecular pathways has been only recently disclosed. This review comprehensively summarizes the most recent knowledge on ATAT1 structure and substrate binding and analyses the involvement of ATAT1 in a variety of cellular processes such as cell motility, mitosis, cytoskeletal organization, and intracellular trafficking. Finally, the review highlights ATAT1 emerging roles in human diseases and discusses ATAT1 potential enzymatic and non-enzymatic roles and the current efforts in developing ATAT1 inhibitors.

## Introduction

Microtubules (MTs), key components of the cytoskeletal architecture in eukaryotic cells, are formed by the polymerisation of α-tubulin and β-tubulin heterodimers in a polar fashion. Most cytoplasmic MTs are composed of 13 protofilaments. However, other protofilament arrangements are also found as in the 9 + 2 protofilament MTs of ciliary axonemes [1, 2]. MT properties and functions are finely regulated by the presence in different MTs of diverse tubulin isotypes and the occurrence of a variety of post-translational modifications (PTMs), giving rise to the so called “tubulin code” [3, 4].

**Fig. 1:**
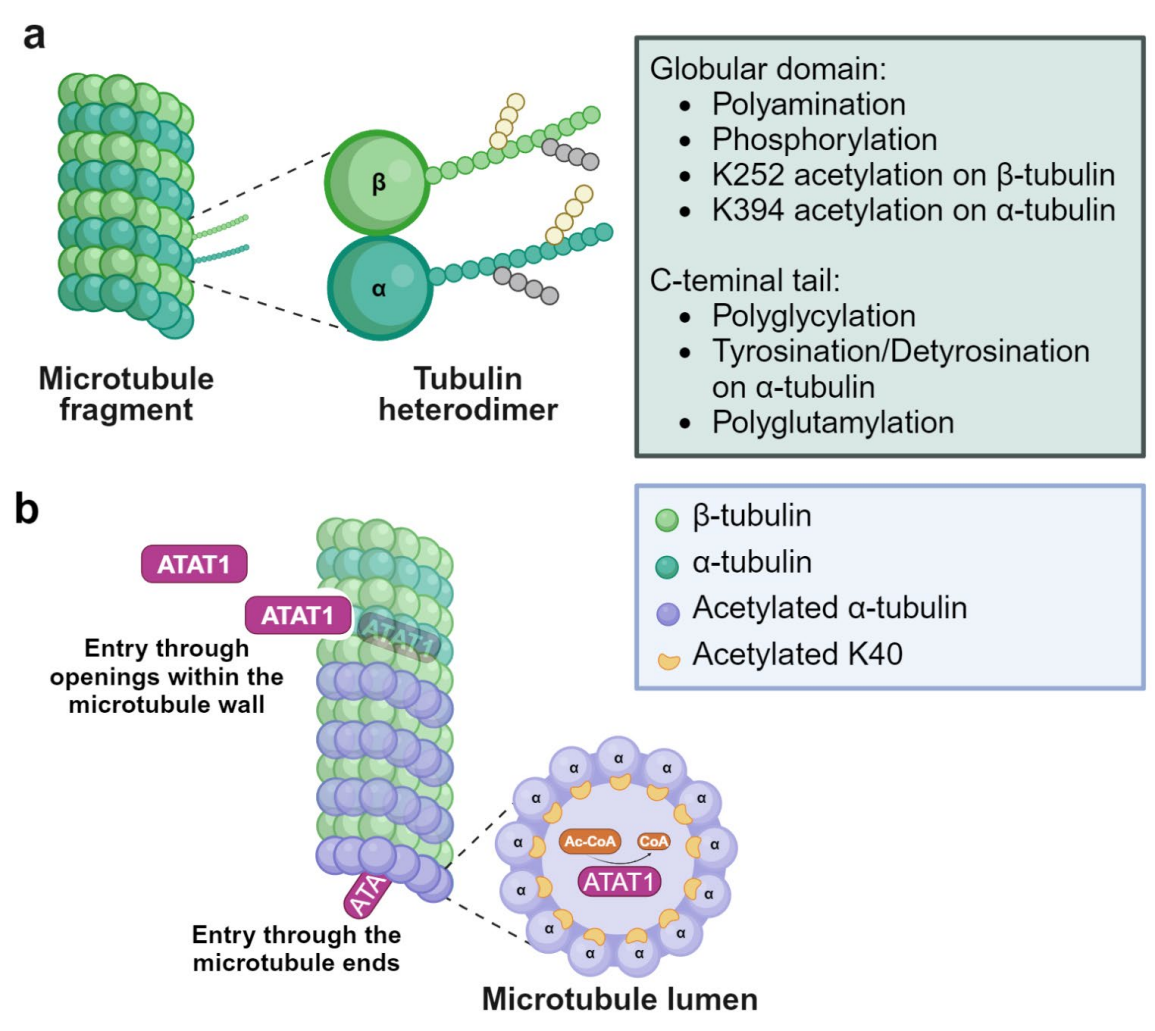
Microtubules, tubulin post-translational modifications and ATAT1. **(a)** Schematic cartoon illustrating microtubule structure and the location of α- and β-tubulin PTMs. Microtubules are tubular structures of α- and β-tubulin heterodimers arranged head to tail to form protofilaments that then associate laterally to form a hollow tube. Tubulin PTMs are located on various positions on α- or β-tubulin. Polyamination, phosphorylation, K252 β-tubulin acetylation and K394 α-tubulin acetylation are detected in the globular part of the protein. Tyrosination/detyrosination, polyglycylation and polyglutamylation chains are found at the *C*-terminal tails of α- and β-tubulin [3, 4]. Other α- or β-tubulin PTMs are not shown. **(b)** ATAT1 is the enzyme responsible for K40 acetylation on α-tubulin. K40 acetylation occurs on the lumen of the tubular structure, where ATAT1 enters through the microtubule ends or through openings within the microtubule wall [40, 41]. Currently, the molecular mechanism of luminal entry is not completely understood. Created with BioRender.com

PTMs occur on amino acids of both tubulin globular domain and C-terminal tail. α- and β-tubulin polyamination and phosphorylation, K252 β-tubulin acetylation and K394 α-tubulin acetylation are detected in the globular part of the protein, with K394 α-tubulin acetylation protruding on the MT surface at the dimer interface. Tyrosination/detyrosination, polyglycylation and polyglutamylation occur on the C-terminal tail of α- and β-tubulin (Fig. 1a) [3, 4]. Proteomic studies have identified 14 acetylation sites in α-tubulin and 12 acetylated residues in β-tubulin [5–8]. Among them, K40, K60 and K370 on α-tubulin and K58 on β-tubulin are luminal residues [8], but only K40 acetylation of α-tubulin has been extensively investigated [9]. Beside K40, K394 on α-tubulin and K252 on β-tubulin are two residues that have been consistently identified acetylated in proteomic studies on various organisms [5–7, 10, 11]. Recently, K394 acetylated α-tubulin has been characterized in the *D. melanogaster *nervous system, where it regulates axonal MT stability during synaptic morphogenesis [12]. K252 acetylation on β-tubulin is catalysed by the San acetyltransferase on soluble tubulin heterodimers. By using acetylation-mimicking β-tubulin mutants in human cultured cells, K252 acetylation has been shown to promote a conformational change that hampers dimer incorporation into MTs and slows down MT polymerisation [6].

To date, acetyl-K40 is the main residue on α-tubulin that has been associated with the presence of long-lived stable MTs [13–15]. Studies with purified acetylated and deacetylated MTs and live cell imaging of MTs have shown that K40 acetylation enhances the flexibility of MTs, giving them more resistance to mechanical breakdown and disassembly [13, 15]. Moreover, this covalent modification has also been linked to the recruitment of specific motor proteins on MTs, affecting intracellular trafficking [16, 17]. Notably, despite the relevance of K40 acetylation in MT functions, studies with non-acetylatable or acetyl-mimicking α-tubulin mutants in various organisms have shown that K40 acetylation is not essential for survival and its absence produces only mild phenotypes [18–23].

In this review, we will focus our attention on the α-tubulin acetyltransferase 1 (ATAT1). Its K40-specific acetylation of α-tubulin renders this enzyme a pivotal player in multiple MT-dependent cell functions. Although several detailed reviews have addressed the role of tubulin PTMs in cellular activities and molecular pathways [24–26], there is a substantial lack of information on ATAT1 with several unanswered questions on its activity. At present, it is still to be clarified whether ATAT1 performs its functions exclusively through the acetylation of α-tubulin or it also acts on different substrates [27]. Moreover, the non-enzymatic activity of ATAT1 remains still undefined [22, 28–31]. This review comprehensively summarizes the most recent knowledge on ATAT1 structure and functions, discusses the evidence supporting the involvement of ATAT1 in various physiological and pathological conditions, and highlights its potential in targeted therapy.

## ATAT1 structure and interaction with microtubules

**Fig. 2:**
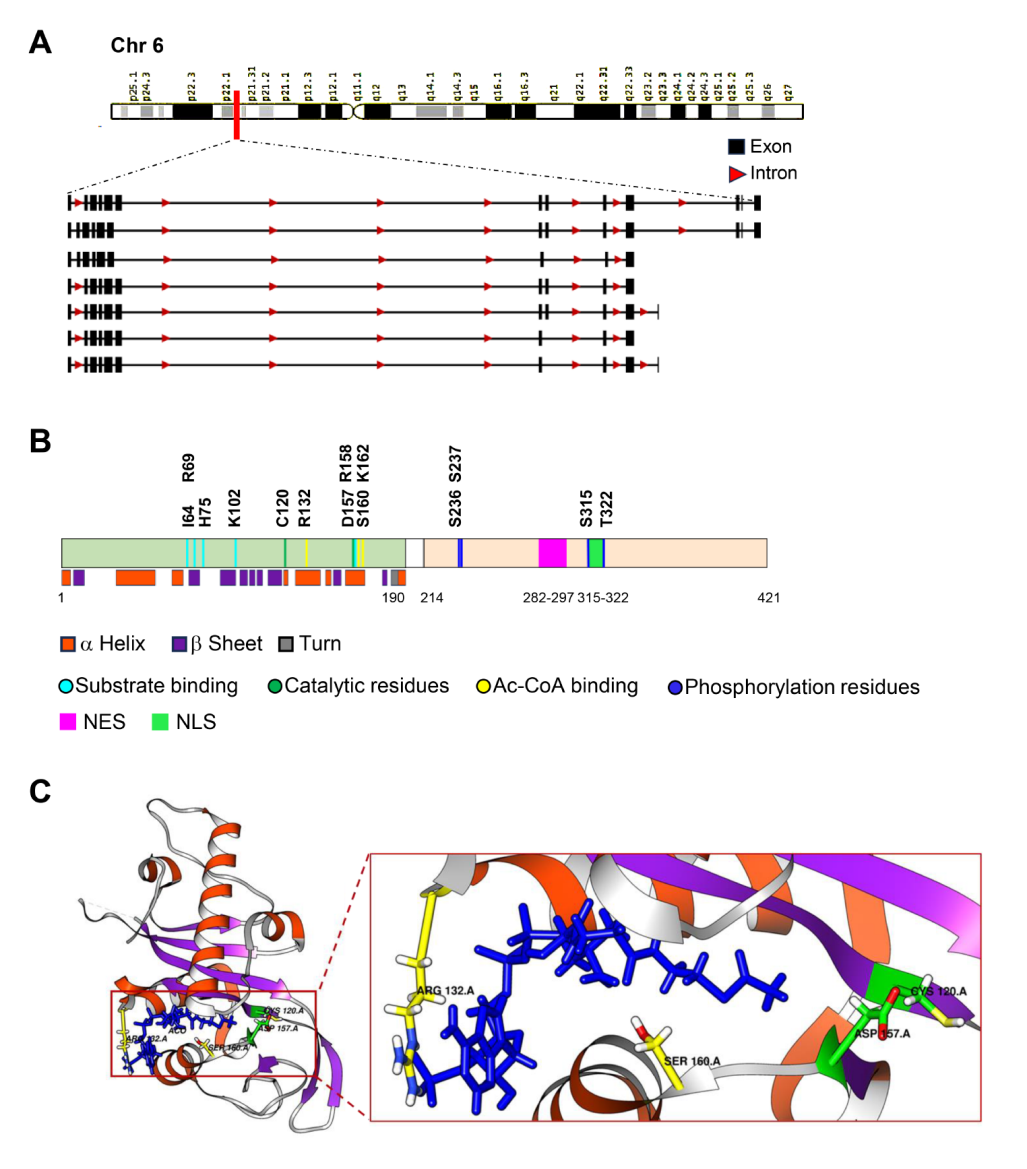
ATAT1 gene locus, protein domains and relevant residues. **(a)** Genomic locus of the human chromosome 6 where the ATAT1 gene maps. Alternative splicing results in 7 transcript variants; the first depicted variant is the canonical transcript that encodes a protein of 421 aa and a molecular mass of 46 kDa. Black boxes indicate exons, red arrows indicate introns (https://www.ncbi.nlm.nih.gov/gene/79969). **(b)** Schematic representation of ATAT1 protein domains. ATAT1 consists of a well characterized *N*-terminal catalytic domain (aa 1-190), and a disordered *C*-terminal domain (aa 214–421) containing a nuclear export signal (NES, aa 282–297, purple) and a nuclear localization signal (NLS, aa 315–322, light green). Residues within the catalytic domain important for activity (green), involved in Ac-CoA (yellow) or α-tubulin binding (turquoise), and relevant phosphorylation sites (blue) are shown. Secondary structure elements composing the catalytic domain are depicted. (orange: α helix; violet: β sheet; grey: turn). **(c)** Crystal structure of the catalytic domain of ATAT1 in complex with Ac-CoA (blue). Ac-CoA is localised within a deep groove in the centre of the catalytic core and makes contacts with different  α helices and   β sheets [37]. Relevant sites for acetyltransferase activity (D157, C120) and Ac-CoA binding (R132, S160) are shown.

In 2010, human ATAT1 was discovered as the homologue of *C. elegans* MEC17 [22, 32], a lysine acetyltransferase related to the GCN5-related N-acetyltransferase (GNAT) superfamily [33]. The ATAT1 gene (also called αK40 TAT, αTAT, MEC-17 or C6orf134) is located on chromosome 6 of the human genome (6p21.33) and consists of 13 exons. Unlike *C. elegans*, no ATAT1 functional paralogues have been described in humans. Beside 421 amino acids and 46 kDa canonical ATAT1, 6 transcript variants, due to alternative splicing, have been identified (https://www.ncbi.nlm.nih.gov/gene/79969, Fig. 2a). The protein is composed of a *N*-terminal catalytic domain, which exhibits homology to other acetyltransferases and a *C*-terminal domain, which was not resolved in crystal structures [34]. The *C*-terminus has been predicted to be intrinsically disordered and to contain a nuclear export signal (NES) and a nuclear localization signal (NLS) [35] (Fig. 2b).

ATAT1 mediates the acetylation of K40 on α-tubulin [32]. Moreover, studies with *wild-type* or non-acetylatable K40 α-tubulin purified from *Tetrahymena* strains have shown that ATAT1 is unable to acetylate other lysine residues on α-tubulin and no recognisable activity has been observed towards purified histones [22]. This substrate specificity is likely to be related to an extensive interaction with α-tubulin that goes beyond the typical enzyme-substrate interaction [36]. To date, the only reported ATAT1 substrate different from α-tubulin is cortactin, an actin-binding protein involved in cell migration and invasion [27].

Several groups have solved the crystal structure of human ATAT1 catalytic domain bound to Ac-CoA (residues 1 to 196) [34, 36–38]. Compared to other lysine acetyltransferases, ATAT1 has a well-conserved co-substrate binding pocket, a distinct active site, and a putative α-tubulin binding site. The identified catalytic domain is composed of a central β-sheet with six antiparallel filaments flanked by three α-helices on each side. The central β-sheet also contains a structural motif called ‘hairpin β’ inserted between β3 and β6, which contacts a loop at the *C*-terminal end of the catalytic domain. The enzyme activity depends upon three conserved phenylalanine residues packed into a hydrophobic pocket within α2 helix. Moreover, 35 amino-terminal residues form a mostly random coiled-coil structure, which maintains Ac-CoA bound to α2 helix in the correct position. Ac-CoA is localised within a deep groove in the centre of the catalytic core and makes contacts with the enzyme via the α2, α4, and α6 helices, the β6 and β7 sheets, and the linker loops, so that ATAT1 makes more than 20 interactions of both hydrophobic and hydrophilic nature with Ac-CoA. The adenine base of Ac-CoA is inserted between K162 and R132 side chains (Fig. 2c). Thus, these two evolutionarily conserved amino acid residues are likely critical for Ac-CoA recognition [36, 37]. Moreover, R132 and S160 participate in stable interactions with CoA and Ac-CoA and are important for ATAT1 protein stability [39].

The application of acetylation assays helped to identify several residues essential in substrate binding and catalysis. D157 and C120 are both required for the catalytic activity of ATAT1. D157 is essential in the organisation of the active site because it aids to form the binding pocket for α-tubulin via a salt bridge. Mutations of this residue lead to enormous structural changes that cause ATAT1 inactivation [34]. ATAT1 is highly specific for the luminal K40 residue of α-tubulin, and R158, I64, R69, H75 and K102 residues on ATAT1 are involved in substrate binding. Indeed, R158 and I64 make the α-tubulin binding pocket hydrophobic and are required to bind and position K40 for acetylation, while R69, H75, and K102 supply the negative charge within the tubulin-binding groove and are required for the binding of the K40-containing α-tubulin loop: mutation of each single residue results in reduced levels of MT acetylation and aberrant substrate binding [37, 38]. The ATAT1 catalytic preference for MTs over free tubulin [32] indicates that the enzyme needs to enter the MT lumen for acetylating α-tubulin. By performing biochemical, fluorescence and electron microscopy experiments on purified ATAT1 and MTs it has been demonstrated that ATAT1 is able to enter the lumen of the tubular structures through the MT ends or through openings within the MT wall (Fig. 1b). Once inside the lumen, the enzyme does not move efficiently inside the MT, being the movement dependent on ATAT1 affinity for its α-tubulin binding sites [40, 41].

Thus, MT structure and ATAT1 affinity for binding sites are important determinants in the acetylation process. To date, it is still unclear whether co-factors/regulators may aid ATAT1 entry and mobility inside MTs, or ATAT1 itself may modulate MT dynamics to promote its access into the lumen.

## Genetic studies of ATAT1 functions in different organisms

A plethora of studies on in vitro cultured cells and in vivo genetic models have identified the role of K40 α-tubulin acetylation in several cellular and developmental processes [9, 26]. Despite this, the contribution of ATAT1 in these processes remains undefined. An overview of the findings obtained in genetic studies on ATAT1 using different model organisms is presented in Table 1.

**Table 1:** **Genetic studies of ATAT1 functions in different organisms**

Model organism	Experimental approach	Phenotype	References
*C. elegans*	MEC17 null and catalytic mutants	control of touch receptor neuron function and MT structure	[22, 31, 32]
	MEC17 null and catalytic mutants, MEC17 overexpression	progressive axonal degeneration, acetylation-independent loss of synaptic branches in neurons	[29, 45]
*T. thermophila*	MEC17 KO	control of microtubule dynamics by K40 acetylation	[32]
*D. melanogaster*	TAT KO and loss-of-function mutants, TAT overexpression	uncoordinated movement, reduced sperm motility, regulation of synaptic bouton growth, impaired touch response	[18, 30, 46, 47]
*D. rerio*	morpholinos against ATAT1	reduced neck and head size, curved body shape and neuromuscular deficiencies	[32]
*Mice*	ATAT1 -/-	impact upon male fertility	[42]
	ATAT1 -/-	ventricular dilation, dentate gyrus deformation, defective motor coordination	[23, 49]
	ATAT1 -/-	migratory and morphological defects in cortical projection neurons	[50]
	ATAT1 conditional KO	defective response to touch and pain	[52]

In the initial studies, deletion of MEC17 in *T. thermophila* and *C. elegans*, TAT in *D. melanogaster* or ATAT1 in mice did not markedly affect organism development or growth [18, 22, 23, 42]. This suggests that both ATAT1 and K40 acetylation are not essential for survival, as suggested by mutation studies of K40 α-tubulin in various organisms [18–23]. Conversely, acetylation-dependent MT functions might also be maintained through redundant or compensating mechanisms.

In *C. elegans*, MEC17 is only expressed in the six mechanosensory neurons, where the protein is required for maintaining a 15-protofilament MT structure typical of mechanosensory neurons in this species [31, 43, 44]. Studies on MEC17 mutants demonstrated impaired touch sensitivity, defective axonal transport and axon degeneration [22, 32, 45]. On the other hand, studies from animals overexpressing MEC17 in the mechanosensory neurons showed a loss of synaptic branches in MT neurons [29]. Furthermore, *C. elegans* strains expressing MEC17 catalytic mutants were shown to have similar frequencies of intact synaptic branches in *wild-type* and mutant animals, indicating that correct levels of MEC17 maintain synaptic branch stability independently from its acetyltransferase activity [29]. These results corroborate the suggestion for a structural role of the protein in MT stability put forward by several studies using MEC17/ATAT1 catalytic mutants in different organisms [28–31, 45].

In *D. melanogaster*, the MEC17 homologue (CG3967, named TAT) was identified as required for larval mechanosensation [18]. Loss-of-function mutants were viable, with no obvious morphological defects but displayed uncoordinated movements, as well as reduced male fertility and sperm motility [46]. During fly development, TAT regulates the larvae locomotion and the growth of synaptic boutons at the neuromuscular junction [30, 47].

*D. rerio* has a single MEC17 homologue, renamed ATAT1. In zebrafish, ATAT1 depletion caused loss of K40 acetylated tubulin and developmental defects such as reduced neck and head size, curved body shape and neuromuscular deficiencies. Notably, ATAT1-knockdown embryos showed a dramatic loss of K40 acetyl tubulin in neurons, but not in cilia, although no ATAT1 paralogues are known in zebrafish [32].

In mammals, ATAT1 functions during development have been studied in mouse knockout models homozygous for ATAT1. Consistent with findings in other organisms, ATAT1 -/- mice were viable and developed normally [23, 42], although decreased sperm motility and male fertility were observed [42]. Interestingly, ATAT1 is highly expressed in differentiating spermatids and in the flagella of elongating spermatids in mice [48], leading to the hypothesis that α-tubulin acetylation in spermatids is relevant for the formation of a normal spermatozoa tail and that α-tubulin acetylation disfunction may be involved in male infertility [48].

Defects in brain development connected to altered neuronal migration were observed in mutant mice and rats [23, 49, 50]. Notably, ATAT1 inactivation led to motor coordination deficits also in knockout adult mice, probably related to a striatal defect [49], and predisposed neurons to axon overbranching and overgrowth [51]. Moreover, ATAT1 inactivation in sensory neurons determined a deficit in mice ability to respond to mechanical touch and pain [52].

Importantly, absence of ATAT1 causes a massive K40 deacetylation of α-tubulin in various organisms and tissues [18, 22, 23, 32, 42], meaning that its function is not fully compensated for by other enzymes. In specific cell contexts, other lysine acetyltransferases belonging to the GNAT superfamily or to the *N*-Acetyltransferase (NAT) family have been shown to acetylate α-tubulin on K40 in mammals, suggesting that these enzymes might have minor α-tubulin acetylation activities [53–56]. In conclusion, ATAT1 is the main K40 α-tubulin acetyltransferase in all species.

## Regulation of ATAT1 expression and activity

**Fig. 3:**
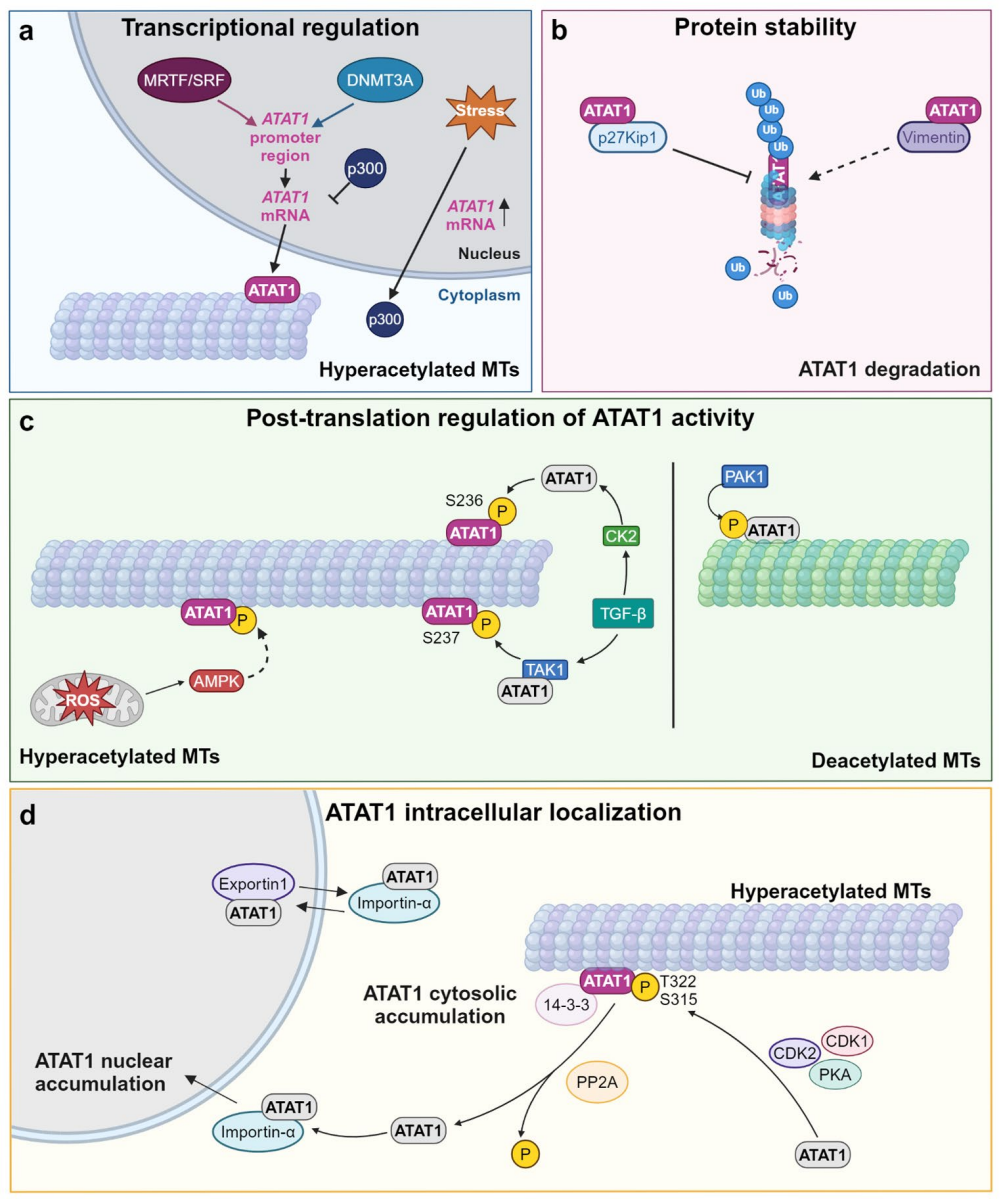
Schematic representation of the different pathways regulating ATAT1 expression and activity. ATAT1 is regulated on numerous levels: **(a) ***Transcriptional regulation*. The MRTF/SRF transcription complex and the epigenetic modifier DNMT3A bind to promoter regions of Atat1 sustaining ATAT1 expression [57]; under normal conditions, ATAT1 mRNA is negatively regulated by the histone acetyltransferase p300 in normal conditions. Following cell stress, p300 is exported from the nucleus to hyperacetylated MTs, relieving its negative regulation on ATAT1 expression [59]. **(b) ***Protein stability.* In neurons, p27Kip1 binds and stabilizes ATAT1 preventing its degradation via proteosome [60]. In cultured cells, vimentin interacts with ATAT1 and enhances its degradation [61]. **(c) ***Post-translational regulation of ATAT1 activity.* Upon stress, activated AMP kinase is suggested to phosphorylate and activate ATAT1 [59]. ATAT1 interacts with TAK1, one of the major TGF-β effectors. TAK1 directly binds and phosphorylates ATAT1 thus promoting its catalytic activation and MT acetylation [64]. CK2, another TGF-β effector, is also required for ATAT1 activation, promoting MT acetylation [65]. In contrast, PAK1 inhibits ATAT1 activity by phosphorylating multiple residues on the ATAT1 *C*-terminal region [66]. **(d) ***ATAT1 intracellular localization.* The intracellular localization of ATAT1 is modulated by phosphorylation on its *C*-terminus. Phosphorylation on T322 and S315 by serine/threonine kinases results in cytosolic accumulation of ATAT1: phosphorylation on these residues prevents NLS interaction with importin-α and promotes ATAT1 binding to specific 14-3-3 adapters. Upon PP2A phosphatase action, dephosphorylated ATAT1 binds importin-α and is transported in the nucleus [35]. Dashed arrow: proposed mechanism. Created with BioRender.com

ATAT1 activity should be finely controlled to achieve the modulation of MT acetylation required in all cellular activities involving cytoskeleton rearrangements; in this context, emerging studies identify upstream and downstream molecular pathways regulating ATAT1 expression and function (Fig. 3). At the transcriptional level, a dramatic down-regulation of ATAT1 mRNA has been observed in retinal epithelial cells devoid of inverted formin 2 (INF2), a formin involved in actin remodelling. The paper identifies the INF2-dependent myocardin-related transcription factor (MRTF)/serum response factor (SRF) transcription complex as controlling ATAT1 transcription by binding the promoter region [57] (Fig. 3a). Furthermore, an epigenetic regulation of ATAT1 expression has been also described. Indeed, by chromatin-immunoprecipitation assays the epigenetic modifier DNA methyltransferase 3 alpha (DNMT3A) was found to bind the promoter region of ATAT1 in bone marrow-derived macrophages and promote mRNA expression. Mechanistically, DNMT3A sustains ATAT1 expression by preventing the association of the histone methyltransferase EZH2 (enhancer of zeste 2 polycomb repressive complex 2 subunit) to the ATAT1 promoter region [58]. ATAT1 mRNA expression is also negatively regulated by the histone acetyltransferase p300 in unperturbed conditions. Following cell stress, p300 is exported from the nucleus to hyperacetylated MTs, relieving its negative regulation on ATAT1 expression [59] (Fig. 3a).

ATAT1 function has also been shown to be controlled by regulated protein degradation. In neurons, the cell cycle regulator p27Kip1 directly binds to ATAT1 and stabilizes it against proteasomal degradation, maintaining MT acetylation and proper MT-dependent intracellular vesicle transport [60]. In line with protein abundance regulating ATAT1 activity, the type III intermediate filament vimentin has been shown to enhance ATAT1 degradation and downregulate α-tubulin acetylation by interacting with ATAT1 [61] (Fig. 3b).

Several studies have shown that ATAT1 activity is regulated by phosphorylation on different residues (Fig. 3c). Upon stress, reactive oxygen species (ROS) are required for MT hyperacetylation through activation of the AMP-activated Protein Kinase (AMPK), which is suggested to phosphorylate ATAT1 [59]. Notably, a further AMPK role in the regulation of ATAT1 expression during stress could not be excluded, as in different cell contexts AMPK phosphorylates and downregulates p300 [62, 63].

Another level of regulation of ATAT1 activity has been identified in the context of the transforming growth factor β (TGF-β) signalling. Here, ATAT1 has been shown to interact with one of the major TGF-β effectors, namely TGF-β-activated kinase 1 (TAK1). TAK1 directly binds and phosphorylates ATAT1 at the S237 position, thus promoting its catalytic activation and MT acetylation to regulate proliferation and metabolism-related pathways [64]. A further paper suggests that S236 phosphorylation on ATAT1 by the TGF-β effector casein kinase 2 (CK2) is required for ATAT1 activation, promoting MT acetylation of fibroblasts grown on soft matrix [65]. Interestingly, correct MT acetylation levels have been found to require p21-activated kinase 1 (PAK1) during platelet formation, a process that requires extensive MT rearrangements [66]. PAK1 has been shown to phosphorylate multiple residues on the ATAT1 *C*-terminal region and inhibit ATAT1 activity in an in vitro kinase assay [66].

A further level of regulation in ATAT1 activity relies on the intracellular localization of the enzyme. Studies of ATAT1 localization in cells are still scanty due to the suboptimal performance of available antibodies. In human fibroblast cells ATAT1 is predominantly cytosolic but it also localizes at the basal body of the primary cilia in quiescent cells and to centrioles and nucleoli in actively growing cells. Throughout mitosis, ATAT1 is observed at the spindle poles and at the midbody, the bottleneck MT structure necessary for cytokinesis. Furthermore, a faint signal on the chromosomes and daughter nuclei is also observed [67]. These localizations are consistent with the binding of ATAT1 to MTs, while the chromosomal localization of the enzyme needs to be better investigated. Recently, time-lapse microscopy in HeLa cells expressing mVenus-ATAT1 has demonstrated that ATAT1 localization is finely tuned by a delicate balance of nuclear export and import dictated by phosphorylation on its *C*-terminus, and that nuclear sequestration of ATAT1 acts to inhibit its MT acetylation activity [35] (Fig. 3d). In the *C*-terminal region, the protein harbours putative NES and NLS signals. Phosphorylation on T322 and S315 by different serine/threonine kinases, such as cyclin-dependent kinase 1 and 2 and protein kinase A results in cytosolic accumulation of ATAT1, whereas nuclear ATAT1 accumulation is favoured by protein phosphatase 2 (PP2A) action. Phosphorylation on T322 and S315 residues prevents NLS interaction with importin-α and promotes ATAT1 binding to specific 14-3-3 adapters, two conditions that enhance ATAT1 accumulation in the cytosol and increase MT acetylation [35]. Based on this work, intracellular ATAT1 localization from regulated nucleus-cytoplasm shuttling may be considered a major mechanism in the regulation of MT acetylation. In conclusion, all these findings suggest that ATAT1 may be controlled both at the transcriptional and post-translational levels to fine-tune its activity in different tissues and under physiological or pathological stimuli.

## ATAT1 in cellular functions

Given that acetylated MTs are intimately involved in multiple cell activities, ATAT1 function may have an impact on organismal physiology and in several diseases, including neurodegeneration and cancer, as discussed below.

### ATAT1 in regulating cell proliferation and mitosis

Although in vivo studies have not identified defective organismal growth in ATAT1-deficient mice lacking α-tubulin acetylation [23, 42, 49], the role of ATAT1 in cell proliferation is still unclear and may be dependent on cell types and growing conditions. Several reports showed that ATAT1 -/- MEFs grew faster than control cells and exhibited loss of contact inhibition [64, 68], and similar results were obtained in cultured mouse fibroblasts [69].

Notwithstanding, ATAT1 silencing in normal pig kidney epithelial cells has been shown to cause severe mitotic effects, mainly characterised by an impaired MT nucleation at centrosomes linked to a defective centrosome recruitment of polo-like kinase 1 (PLK-1), a major spindle assembly kinase. In addition, ATAT1 silencing was found to result in an impaired interaction between kinetochores and MTs, possibly by producing breaks into the MT bundles interacting with kinetochores [70]. These findings suggest that acetylation of MTs by ATAT1 regulates mitotic spindle plasticity and integrity.

A relevant question is whether ATAT1 effects on cell cycle progression are connected to its influence on MT dynamics. In a recent study, Lopes and coauthors have shown that ATAT1 silencing has no significant effects on the turn-over of mitotic MTs and does not affect MT growth in interphase cells, suggesting that MT dynamics is unperturbed when tubulin acetylation is impeded [71]. These findings, together with the reported effects on mitotic MT integrity [70], could be explained with the proposed action of K40 acetylation and ATAT1 in enhancing MT flexibility and increasing resistance to MT breakage [13, 15]. These functions could be highly relevant in the control of mitotic progression and fidelity.

Primary cilia are post-mitotic cellular organelles detecting extracellular cues [72]. Although α-tubulin acetylation is recognized as an important hallmark of ciliated structures, the role of ATAT1 in ciliogenesis is still controversial. In mice and human cultured cells, ATAT1 knockdown does not abrogate primary cilium formation [23, 42] but alters the normal kinetics of ciliogenesis [22, 73]. Notably, from a molecular point of view ATAT1 catalytic activity has been shown to control non-muscle myosin IIB (Myh10) expression to regulate ciliogenesis kinetics [73].

### ATAT1 in cell migration

Cell migration is essential in a broad range of biological processes, including development, immunity, and cancer [74]. Recent evidence suggests that tubulin acetylation and ATAT1 play a crucial role in in vitro and in vivo cell migration [49, 75, 76]. In primary astrocytes, ATAT1 and acetylated MTs have been found to preferentially localize at focal adhesions, thereby regulating cell migration. Indeed, inhibition of ATAT1 expression, leading to decreased K40 tubulin acetylation, reduced the speed of cell migration without affecting migration direction or whole cell movement [75]. In the same study, ATAT1 was found to regulate focal adhesion turnover by promoting the fusion of Rab6-positive vesicles at focal adhesions [75]. Recent evidence shows that, in conditions of substrate rigidity, ATAT1 is recruited to focal adhesions by a talin- and actomyosin-dependent mechanosensing signalling for matrix stiffness-dependent microtubule acetylation [76]. Overall, these studies support the promigratory role of ATAT1-dependent tubulin acetylation in neuronal tissues.

The link between ATAT1 function and migration is corroborated by a further study using human dermal fibroblasts (HDFs). When grown on stiff substrates, HDFs exhibit higher levels of acetylated tubulin and show a greater ability to contract and migrate as compared with cells on soft substrates. Following ATAT1 depletion, HDF contraction and migration were markedly decreased in stiff conditions, concomitantly with a decreased expression of extracellular matrix proteins playing a key role in skin fibrosis [77]. Mechanistically, ATAT1 has been shown to influence the cellular response to matrix stiffness by regulating the phosphorylation of the mechanosensitive protein YAP and controlling its nucleocytoplasmic shuttling [76, 77]. Contrasting results are available on the role of ATAT1 in migrating macrophages [58, 78], whereas live cell imaging of ATAT1-depleted human epithelial cells did not detect any alteration in directed cell migration, cell polarization and cell length in a 1D migration assay [79]. Thus, ATAT1 role in cell migration is still unclear and is likely to depend on cell types and tissue conditions.

### ATAT1 in intracellular trafficking

Intracellular trafficking is a tightly regulated MT-dependent process that moves vesicles and substances within the cell [80]. Different studies have shown that MT acetylation promotes the recruitment of both dynein and kinesins to MTs, thereby enhancing bidirectional intracellular trafficking [16, 17, 81]. In neurons, ATAT1-dependent MT acetylation has been linked to the axonal transport of vesicles and organelles [60, 82]. Notably, it has been demonstrated that ATAT1 is transported at the cytosolic side of neuronal vesicles moving along axons, suggesting that this axonal transport of ATAT1-enriched vesicles is the predominant driver of tubulin acetylation in axons [82].

ATAT1 is also implicated in autophagy, a membrane-trafficking pathway that delivers cellular components to the lysosomes for degradation and recycling [83]. ATAT1 silencing has been shown to promote or impair autophagosome maturation in different cellular contexts, so that further studies are needed to better clarify its role in the autophagic process [84, 85].

Several lines of evidence indicate that tubulin acetylation and ATAT1 are also involved in the movement along MTs of cell organelles, such as mitochondria, lysosomes, endosomes, or lipid droplets [81, 86–89]. Recently, ATAT1 has been found in a multiprotein complex containing GCN5-like 1 (GCN5L1) protein and RAN-binding protein 2 (RANBP2) in hepatocytes, where the complex controls lysosome positioning in the cells [86].

Alcohol consumption has been shown to induce MT acetylation in hepatocytes, which, in turn, leads to the accumulation of large, immobile lipid droplets, storage organelles at the centre of lipid and energy homeostasis. Notably, ATAT1 overexpression in unperturbed conditions was found to enhance dynein binding to large lipid droplets, thereby leading to impaired droplet dynamics [87]. Furthermore, acetaldehyde exposure and ATAT1 overexpression significantly impaired other MT-dependent processes, such as protein secretion and transcytosis, and clathrin-mediated endocytosis in cultured hepatocytes [88].

Recent evidence has shed light on the role of ATAT1 and K40 tubulin acetylation in the global reorganization of organelle intracellular positioning that occurs following centrosome amplification, a condition commonly observed in cancer. In cells with supernumerary centrosomes, several cell organelles are displaced towards the cell periphery and the same is found for the intermediate filament marker vimentin. In these cells, centrosomes, mitochondria, and vimentin displacement has been found to be dependent on ATAT1-mediated MT acetylation that would favour kinesin-1-dependent organelle movement. In contrast, endosomes and Golgi displacement was unaffected by ATAT1 depletion, suggesting that other PTMs or protein adaptors could mediate the relocation of these organelles [81]. Collectively, these data highlight a complex relationship between intracellular trafficking, MTs, and α-tubulin acetyltransferase activity that needs further investigation.

### ATAT1 in the response to cellular stress

There is growing evidence that MTs and tubulin PTMs play a critical role both in sensing and responding to cell stress [90]. Indeed, changes in tubulin acetylation status appear as a reversible cell stress response to different stressors; however, the role of ATAT1 in stress response is still understudied. Using ATAT1 -/- MEFs, Yang and collaborators have proved that ATAT1 is a crucial effector of stress-induced α-tubulin hyperacetylation [49]. Interestingly, another study has delineated a role for p300 and AMPK as mediators of ATAT1 activation upon high salt-induced cell stress [59].

A more recent report has identified a role for ATAT1 in the response to genotoxic stress. ATAT1 silencing in HeLa cells reduced DNA damage-induced checkpoint activation in response to the topoisomerase I inhibitor camptothecin. Notably, expression of a wild-type ATAT1 but not of a catalytically inactive protein rescued the checkpoint defect observed in ATAT1 depleted cells, suggesting that ATAT1 impacts on the DNA damage checkpoint via an acetylation-dependent mechanism [91]. Interestingly, silica exposure and matrix stiffening or a combination of both lead to a downregulation of ATAT1-dependent tubulin acetylation with subsequent DNA damage and replication stress in a rat model of silicosis and in a lung cancer cell line [92], confirming a relationship between ATAT1 and DNA damage response.

ATAT1 and MT acetylation also tunes cytoskeletal stiffness, which affects cytoskeletal mechanics and mechano-transduction in specialized cells. In striated muscle, overexpression of ATAT1 increases viscoelastic resistance and dampens contractile kinetics [93]. In astrocytes and endothelial cells, ATAT1 is required for the mechanosensitive regulation of migration [76]. In a rat model of lung silicosis, a fibrotic condition resulting in cell stiffening, a reduced expression of ATAT1 was observed in silicosis nodules or interstitial fibrotic regions along with an increased fibroblast-to-myofibroblast differentiation. ATAT1 down-regulation could be reversed by an anti-fibrotic tetrapeptide, implicating ATAT1-dependent tubulin acetylation as part of an anti-fibrotic mechanism [94].

## Emerging roles for ATAT1 in neurological diseases and cancer

In brain tissues, the observed high levels of ATAT1 and of K40 acetyl α-tubulin have been linked to the need of efficient MT-based transport of molecules and organelles in the extended axonal cytoplasm [23, 45, 60, 82, 95, 96]. In line with this, increasing evidence demonstrates the role of ATAT1 function in some neurodegenerative diseases. Studies using β amyloid (Aβ)-secreting cell lines expressing Alzheimer’s disease (AD) mutations, as a cellular model for the disease, demonstrated that the increased MT acetylation and Aβ secretion stimulated by H_2_O_2_-induced oxidative stress were dependent on ATAT1 activity [97]. Concordantly, ATAT1 knockdown in the hippocampal region of an AD mouse model produced significant reductions in Aβ plaque accumulation in the cerebrospinal fluid and restored the memory loss, characteristic of this disease model [97]. Overall, these results highlight a link between ATAT1-dependent MT acetylation in response to oxidative stress and amyloid plaque formation in the early stages of AD. Interestingly, inhibition of ATAT1 expression by depletion of the epigenetic modifier METTL3 resulted in alleviated AD symptoms in a disease mouse model by reducing α-tubulin acetylation and enhancing brain migration of monocyte-derived macrophages involved in Aβ plaque clearance [58].

Charcot–Marie–Tooth disease type 2 A (CMT2A) disease is a peripheral neuropathy resulting from mutations in the mitochondrial fusion protein mitofusin-2 (MFN2), a large GTPase implicated in mitochondrial fusion and tethering with the endoplasmic reticulum [98]. A recent paper has shown that the sites of mitochondrial contacts with MTs are regions of high -α-tubulin acetylation which is mediated by the MFN2-dependent recruitment of ATAT1 at these sites, and that this function is important for MFN2-dependent control of mitochondria motility [89]. The findings in the paper suggests that a defective ATAT1-dependent MT acetylation may underly the impaired axonal mitochondrial mobility observed in CMT2A disease [99].

Leucine-rich repeat kinase 2 (LRRK2) mutations are the most common genetic cause of Parkinson disease (PD) [100]. The mutated LRRK2 protein accumulates in filamentous structures that associate with deacetylated MTs, inhibiting axonal transport and causing locomotor deficits in *Drosophila* LRRK2 mutants. Increasing MT acetylation by deacetylase inhibition prevented the formation of filamentous LRRK2, restored axonal transport in rat cortical neuron cultures and rescued the transport and locomotion deficits in a *Drosophila* model of the disease. Interestingly, ATAT1 expression also prevented the formation of the mutant LRRK2-dependent filamentous structures [101].

Aberrant levels of tubulin K40 acetylation have been reported in different cancer types [9, 90, 102–105]. In the last years, many studies have highlighted the involvement of ATAT1 functions in breast tumours [27, 105–107], while ATAT1 role in other tumours remains still elusive (Table 2**)**.

**Table 2:** ATAT1 studies in cancer

Cancer Type	Experimental Approach	Impact on Cancer Cell Properties	References
Breast cancer	Overexpression	control of endosome localization and dynamics, microtentacle formation, promotion of migratory and invasive capacities	[27, 105]
	Downregulation	induction of ER stress and ECM degradation, inhibition of cell migration, invasion, proliferation, and spheroid formation, attenuated tumor growth	[27, 106, 107]
Hepatocellular carcinoma	Downregulation	in vitro and in vivo sensitization to a novel MT-targeting drug	[102]
Ameloblastoma	Downregulation	inhibition of cell migration and invasion, no effect on cell proliferation	[108]
Colon Cancer	Downregulation	inhibition of proliferative and invasive cell activities, no impact on paclitaxel cytotoxicity	[71, 109]
Cervical cancer	Downregulation	mitotic catastrophe, defective DNA damage-induced checkpoint activation	[91, 110]
Lung Cancer	Overexpression	attenuated cell motility and invasion, enhanced metastatic ability, in vivo paclitaxel resistance	[104, 111].
	Downregulation	promotion of cell migration and invasion, DNA damage and replication stress, inhibition of cell proliferation and polyploidy induction, autophagosome formation	[84, 92, 110, 111]

In breast cancer, tubulin acetylation and ATAT1 have been found to regulate cell protrusions, adhesion and invasion, key steps of tumour dissemination. In particular, ATAT1 overexpression in a non-metastatic cancer cell line increases K40 tubulin acetylation and enhances the formation of microtentacles, membrane-based protrusions that aid cells in suspension to reattach to the substrate [105], while in a metastatic cancer cell line ATAT1 colocalizes and regulates cortactin acetylation levels, and this colocalization is required for the migratory and invasive capacities of these cells [27]. Silencing of ATAT1 also promotes the accumulation of endosomes containing membrane-type I matrix metalloproteinase (MT1-MMP) at peripheral adhesion sites, suggesting that tubulin hypoacetylation may favour MT1-MMP extracellular delivery for enhancing matrix degradation [27]. In another breast cancer study, ATAT1 catalyses MT acetylation at the membrane-associated clathrin-coated pits through its interaction with the clathrin adaptor AP2 and ATAT1/AP2 interaction is required for directional migration [106]. In cells grown on a stiff matrix, ATAT1 silencing induces ER stress and inhibits cell migration, invasion, proliferation, and spheroid formation, via downregulation of gene expression related to cancer-related pathways [107]. Moreover, analysis of ATAT1 transcripts in breast cancer databases has shown that ATAT1 is upregulated in most cancer tissues when compared to the normal tissues [107]. These findings are consistent with the observation that increased levels of tubulin acetylation correlates with progression and poor prognosis in breast cancer patients [105]. Overall, these studies suggest that ATAT1 may be considered an important molecular marker of invasion and progression in breast cancer.

Interestingly, ATAT1 downregulation also inhibits cell invasion and migration in colon and ameloblastoma cancer cells and induces mitotic catastrophe in lung cancer cells [108–110] In contrast, ATAT1 overexpression inhibits cell migratory and invasive ability of lung cancer cells as well as in vivo tumour metastasis [111].

Recent studies have examined the correlation between tubulin acetylation levels and sensitivity to microtubule drugs. While one study showed that ATAT1 silencing did not impact paclitaxel cytotoxicity in colon cancer cells [71], a correlation between high tubulin acetylation levels and paclitaxel resistance was observed in patient-derived lung cancer cells and in paclitaxel-resistant lung cell lines [104]. Interestingly, ATAT1 overexpression was found to mediate paclitaxel resistance by preventing the degradation of Mcl-1, an anti-apoptotic protein involved in paclitaxel resistance [104]. Recently, ATAT1 downregulation has been reported to reduce K40 acetyl tubulin levels and sensitize hepatocellular carcinoma cells to a novel MT-targeting drug [102]. Overall, these studies show that altered α-tubulin acetylation is linked to various human diseases and that ATAT1 may constitute a novel target for drug development and therapeutic intervention.

## Recent advances in the development of ATAT1 inhibitors

**Fig. 4:**
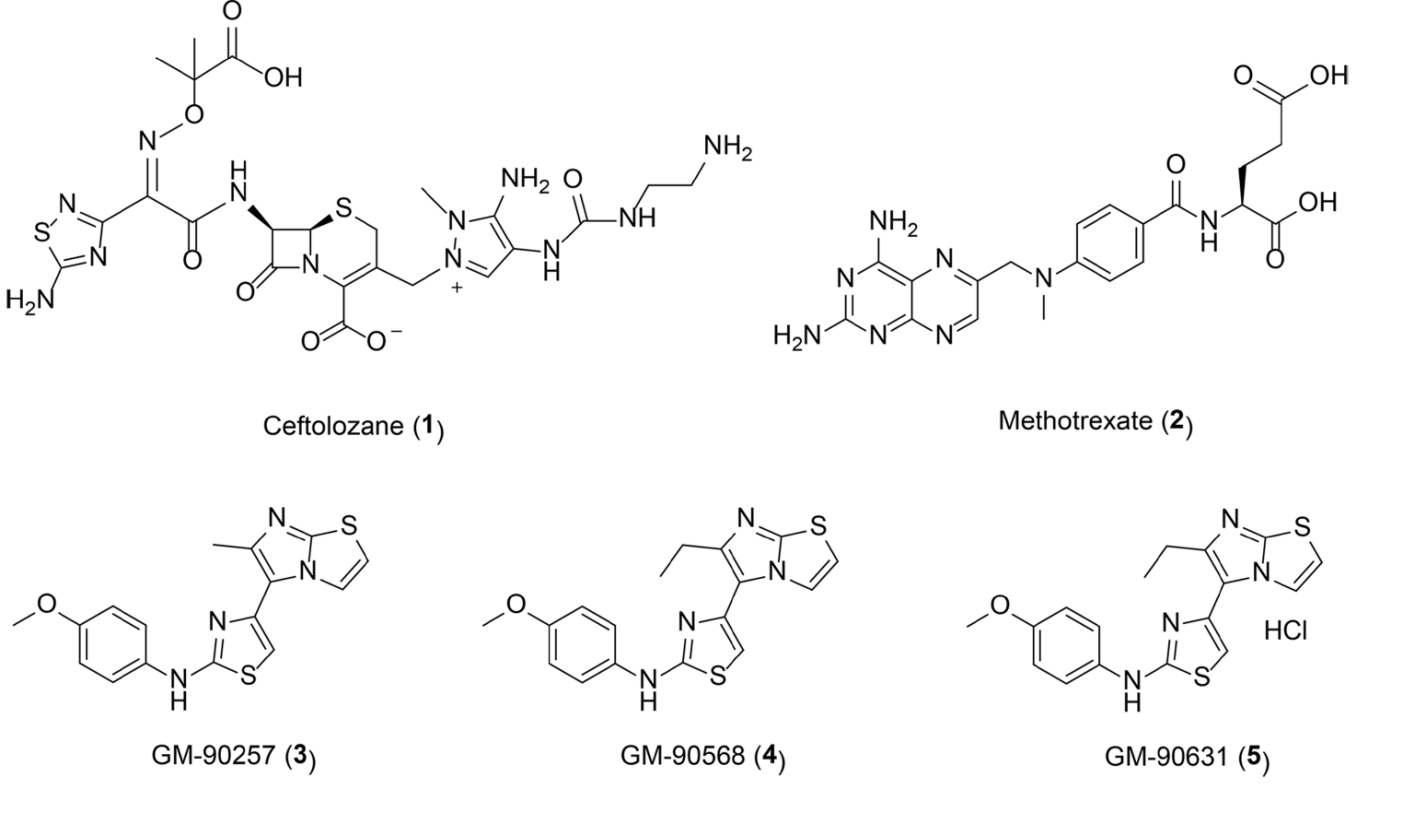
Proposed ATAT1 inhibitors Chemical structures of ceftolozane (**1**) and methotrexate (**2**) predicted as high-energy binders of ATAT1 [112]. Chemical structures of a series of 2,4-disubstituted thiazole compounds (**3–5**) identified as inhibitors of microtubule acetylation in vitro, in TNBC cells and mouse xenograft models [103]

To the best of our knowledge, only a couple of studies dealing with potential ATAT1 small-molecule inhibitors have been reported so far. In 2021, Yang and collaborators proposed two models of ATAT1 which recapitulate the structural features that the molecules must have for anchoring to the target (indicated as “pharmacophore anchor models”). The authors developed these models according to the structural conformations induced by the ATAT1 natural ligands Ac-CoA and CoA, identified in the analysis of 14 crystal structures of ATAT1 from different protein data banks [112]. These models were statistically constructed using thousands of docked compounds, and allowed to infer the structural moiety preferences with the physico-chemical properties for the binding to the ATAT1 active site. In particular, the proposed pharmacophore anchor models revealed 3 sub-pockets S1 (acetyl site), S2 (adenine site), and S3 (diphosphate site) with 8 anchors where conserved moieties interact with respective sub-pocket residues in each site and helped in guiding ATAT1 inhibitor development. Indeed, once completely validated, the authors applied the model in a virtual screening and predicted 10 potential inhibitors and their binding mechanisms. Despites some of the predicted high-energy binders such as ceftolozane and methotrexate (**1, 2** in Fig. 4) are known drugs with a great repurposing potential, none of them has been investigated in in vitro inhibition assays with ATAT1 [112].

In 2020 Song and collaborators reported a series of 2,4-disubstituted thiazole compounds (**3–5** in Fig. 4) as inhibitors of MT acetylation in triple-negative breast cancer (TNBC) cells and mouse xenograft models [103]. After a preliminary cell-based screening of a library of about 30,000 small-molecule compounds, the authors identified GM-90,257 (**3**) as a first hit able to reduce acetyl-α-tubulin levels in breast cancer cells at submicromolar concentrations in a dose-dependent fashion. The optimization of **3** led to the very similar superior homologue GM-90,568 (**4**) and to its hydrochloride salt GM-90,631 (**5**) that resulted able to reduce K40 α-tubulin acetylation and disrupt the MT structure in MDA-MB-231 cells at low concentrations (50–100 nM) without affecting α-tubulin expression and histone H3 acetylation. Moreover, the authors observed a decrease of acetyl-α-tubulin levels at pharmacological doses together with a significant reduction of tumor growth in vivo in mouse xenograft TNBC models after intraperitoneal administration of the chemical. Extensive docking simulations performed on enzymes involved in MT acetylation (HDAC6, SIRT2, ATAT1) led the authors to exclude a direct interaction of **3–5** with the active site of these enzymes, while supported their direct binding to the K40 residue of α-tubulin that should prevent ATAT1 from binding to MTs. Despite the proposed mechanism of inhibition as disruptors of the interaction between α-tubulin and ATAT1 should be confirmed by additional more informative experiments, this series of compounds define the only validated chemotype for MT acetylation inhibition available so far.

## Open questions and concluding remarks

In the recent years, exciting new links between tubulin acetylation, ATAT1 and a range of cellular functions have been discovered, but several questions remain unanswered. Firstly, it is still to be determined whether ATAT1 can acetylate residues different from K40; indeed, the lack of antibodies capable of detecting other acetylation sites makes it difficult to determine whether K40 of α-tubulin is the only target of ATAT1 on MTs or whether other luminal residues (e.g. K60 or K370) may also be targeted. Furthermore, the functional interaction between ATAT1 and San, the acetylase that modifies K252 on β tubulin, as well as the interplay of ATAT1 with other minor α-tubulin acetylases or with the different tubulin deacetylases is far from being uncovered. Next, the only other protein identified as ATAT1 target so far is cortactin [27], although several mechanistic aspects remain to be explored, including the residue/s that is acetylated and the ATAT1 binding site on the protein. Nevertheless, this study suggest that the number of ATAT1-targeted proteins may increase in the future, opening the possibility for tubulin-independent ATAT1-connected functions. Finally, a kinase-mediated regulation of ATAT1 nuclear export and import has been reported [35]. According to these findings, nuclear localisation of ATAT1 restricts its access to polymerised MTs, thereby finely regulating tubulin K40 acetylation. However, it remains to be clarified whether ATAT1 has also specific function/s once in the nucleus. There, it may take part in multi-protein complex/es, as found in [86], and acetylate as yet unidentified substrates, thereby contributing to key functions in DNA replication and repair processes. Thus, exploring new ATAT1 substrates is a great challenge for the coming years, and will certainly contribute to uncover novel biological pathways regulated by this enzyme.

Interestingly, the K40 residue of α-tubulin is also targeted for tri-methylation (K40me3) by SETD2, a dual-function histone and microtubule methyltransferase [113]. K40me3 is associated with polymerized MTs in cells and promotes MT stabilization in vitro [113, 114]. Furthermore, it has been observed that α-tubulin K40me3 is increased in the absence of K40 acetylation, and that this increase rescues the defects in radial migration and morphological transition of cortical neurons caused by ATAT1 depletion [114]. These results suggest that the two modifications may have overlapping effects, but it is still to be determined whether the two enzymes compete to access the K40 residue and their functional interactions in specific cellular events.

Finally, several reports have indicated acetylation independent ATAT1 activities in different organisms, mostly in neuronal tissues suggesting that ATAT1 might exert also structural functions in neuronal cell harbouring extended axonal MTs [30, 32, 42]. Both functions may contribute to maintain a functional MT network both by local enzymatic activity and by promoting ATAT1 accumulation on long-lived MTs. Structural/signalling ATAT1 activities appear to be relevant during neuron development and are likely to be important in neuronal aging and neurodegenerative diseases [30, 50]. Nevertheless, the importance of non-enzymatic activities in other ATAT1-related functions, including cell migration and non-axonal intracellular transport, as well as in non-neuronal diseases such as cancer, are still to be clarified.

Despite recent evidence which points to ATAT1 and K40 tubulin acetylation as significant players in several types of cancer, in particular breast cancer [90, 102–106], little is known about the underlying mechanisms and the relevance of ATAT1 acetyltransferase activity in the cancer process. Further investigations are needed to better clarify the pathological role of ATAT1 in cell transformation and cancer progression, in which the identification of novel targets and pathways regulated by ATAT1 might represent an important advance toward a more personalized treatment of human tumours.

## Data Availability

Not applicable.
